# Lectin-Grafted PLGA Microcarriers Loaded with Fluorescent Model Drugs: Characteristics, Release Profiles, and Cytoadhesion Studies

**DOI:** 10.3797/scipharm.1312-08

**Published:** 2014-02-08

**Authors:** Xue-Yan Wang, Romana Koller, Michael Wirth, Franz Gabor

**Affiliations:** Department of Pharmaceutical Technology and Biopharmaceutics, University of Vienna, Althanstrasse 14, 1090, Vienna, Austria.

**Keywords:** Caco-2, Cytoadhesion, Microparticles, PLGA, Wheat Germ Agglutinin (WGA)

## Abstract

PLGA microparticles loaded with three different fluorescent model drugs, fluorescein sodium (hydrophilic), sulforhodamine (amphoteric), and boron-dipyrromethene (BODIPY^®^ 493/503, lipophilic), were prepared by the solvent evaporation technique. Due to varying hydrophilicity, the diameters of the microparticles ranged between 4.1 and 4.7 μm. According to fluorimetric analysis, the loading varied from 0.06 to 2.25 μg of the model drug per mg PLGA. In terms of the release profile, the fluorescein sodium-entrapped formulation exhibited thermo-responsive release kinetics. In the case of sulforhodamine- and BODIPY^®^ 493/503-loaded particles, almost no release was observed, neither at 4°C nor 37°C during the first 50 hours. Furthermore, to estimate the bioadhesive properties of such drug delivery systems, the surface of the loaded particles was grafted with wheat germ agglutinin by applying the carbodiimide method. Cytoadhesion studies with Caco-2 monolayers revealed an up to 1.9-fold and 3.6-fold increase in the bioadhesion of the lectin-functionalized, model drug-loaded particles as compared to the albumin- and non-grafted microcarriers, respectively. All in all, the results clearly indicated that the lipophilicity of the polymer matching that of the drug favored entrapment, whereas mismatching impeded loading into the PLGA-microparticles. Even in the case of low loading, these delivery systems might be useful for the fluorescent detections and microscopic imaging of cellular interactions due to their fluorescent properties and lack of dye leakage. Moreover, lectin grafting can mediate bioadhesive properties to such particulate drug carriers which could be a promising approach to improve drug delivery.

## Introduction

As is nowadays generally accepted, the administration of polymeric micro- and nano-carriers as particulate drug formulations offers numerous advantages in comparison to conventional approaches, such as the protection of encapsulated active pharmaceutical ingredients (APIs) against the harmful gastrointestinal environment, the possibility of encapsulating hydrophilic as well as lipophilic drugs, the controlled release of loaded APIs, the feasibility of different physicochemical targeting approaches, as well as the improvement of bioavailability [1]. Because of these reasons, the characteristics of numerous polymeric drug carriers have been intensively studied. Among these polymers, the most commonly investigated one is PLGA (poly(lactic-co-glycolic acid)) due to its biocompatibility and biodegradability, its ability to modify the release profiles by use of different polymerization grades, as well as its long-time use in humans resulting in being “generally recognized as safe” by the FDA [2–6]. In terms of encapsulation of APIs with poor biopharmaceutic and pharmacokinetic properties, a wide variety of drugs has been successfully applied in particulate delivery [2], such as the antioxidants coenzyme Q10 [7] and curcumin [8], the anti-leishmanial agent amphotericin B [9], the immunosuppressant cyclosporine [10], the anti-cancer agent doxorubicin [11], and others. In the case of surface modification for drug targeting, the uncapped types of PLGA with terminal carboxylate groups provoked interest due to the feasibility of functionalization with a number of ligands, especially proteins and peptides. In particular, the surface modification of particles with lectins is a promising approach for gastrointestinal targeting [12]. In order to prolong the residence time in the GI tract by improving mucoadhesion, immobilization of wheat germ agglutinin (WGA) from *Triticum vulgare* might be useful. Due to its specific interaction with sialic acid and the N-acetyl-D-glucosamine moieties of the glycocalyx of enterocytes [13], several drug delivery systems functionalized with WGA have been developed in our group for intestinal targeting [14–17].

In the present work, three different fluorescent dyes were entrapped into PLGA microparticles. Fluorescein sodium, sulforhodamine, and 4,4-difluoro-1,3,5,7,8-penta-methyl-4-bora-3a,4a-diaza-*s*-indacene (BOD; BODIPY^®^ 493/503) were chosen as model drugs due to their differing solubilities being hydrophilic, amphoteric [18], and lipophilic, respectively. PLGA-microcarriers were prepared using the solvent evaporation technique either as water-in-oil-in-water (double emulsions) or as oil-in-water (single emulsion) methods according to the lipophilicity of the loaded fluorescent dyes. Moreover, the surface of the microparticles was modified with ligands (e.g. WGA) by the carbodiimide method, which mediates the formation of amid linkages between carboxylic and primary amine groups. Therefore, instead of the commonly used stabilizer polyvinylalcohol (PVA), poly(ethylene-*alt*-maleic acid) (PEMA) was applied. Due to its carboxylate content PEMA might enhance the coupling efficiency as described by Keegan et al. [19]. Subsequently, the size, stability, payload, and release profile of the loaded model drugs of the prepared microparticles were analyzed. Additionally, the surface of the microcarriers was modified to mediate specific bioadhesive properties. In comparison to the albumin- and non-grafted drug carriers, the impact of this functionalization was examined *in vitro* with Caco-2 cell monolayers, which is a generally accepted and well-established epithelial cell model [20].

## Results and Discussion

### Characterization of the Model Drug-Loaded PLGA-Microparticles

Due to its hydrophobic characteristics, BODIPY^®^493/503 was entrapped into micro-particles via the single emulsion (o/w) method, whereas fluorescein sodium as well as sulforhodamine were encapsulated applying the (w/o/w) double emulsion technique. As illustrated in Table 1, independent from the type of loaded model drug, the particles possess a comparable mean diameter of about 4.4 μm. There are some reasons rendering this size range appropriate for the intestinal drug delivery: first, in contrast to nanoparticles, the total API-loading is higher. Second, at the same weight, particles of low micron size provide a larger surface area for functionalization with targeters than larger particles. Third, according to Desai et al. [21], particles in the low micrometer size range are not likely to be taken up by the cells, but rather remain in the intestinal lumen. Therefore, such particles could facilitate a long-term drug release in a controlled manner. Furthermore, in order to enhance the capability of later covalent conjugation with primary amine-bearing protein ligands to the particle surface by carbodiimide chemistry, PEMA (poly(ethylene-*alt*-maleic acid)) as a new stabilizer instead of the traditional one poly(vinyl alcohol) (PVA) was applied by the solvent-evaporation technique [19].

Among all microparticle preparations, the BOD-loaded microparticles exhibited the smallest mean particle size, the highest model drug loading, and the highest encapsulation efficiency (Table 1). This might be due to the lipophilicity of both, the dye and the polymeric matrix, which results in high affinity between both components. As a result of these prevailing strong hydrophobic interactions, the fluorescent dye preferentially remains in the oily phase together with PLGA during the emulsification step as reflected by the high dye loading and also yields tighter and smaller microparticles by the single emulsion technique [22, 23]. In contrast, the dye loading of fluorescein sodium-loaded microcarriers was 22% lower. This result is due to low affinity between the hydrophilic dye and the hydrophobic polymer. Consequently, in the case of the double emulsion technique, hydrophilic drugs can easily distribute from the inner aqueous phase to outer aqueous medium during the emulsification process, which considerably reduces the loading efficiency. Interestingly, although the fluorescein sodium and sulforhodamine-loaded particles were prepared by the same technique, and equimolar amounts of dyes were applied, the encapsulation efficiency of these two model drugs varied considerably. As demonstrated in Table 1, the entrapped amount of fluorescein sodium was approximately 1.75 μg per mg PLGA, in contrast to 0.06 μg per mg PLGA in the case of sulforhodamine. This might be due to the amphoteric character of sulforhodamine, which allowed the red fluorescent substance to accumulate inside the aqueous phase as well as inside the oil phase during the first emulsification step. Therefore, after the addition of the second aqueous phase, sulforhodamine might have been distributed into this external phase and washed out during the subsequent purification step.

Furthermore, after the surface modifications with the targeters, all the microparticles were homogenously dispersible in buffer, and stored stably at −80°C for at least one month until further experiments.

### Release Kinetics of the Entrapped Fluorescent Model Drugs from PLGA Microparticles

**Fig. 1:**
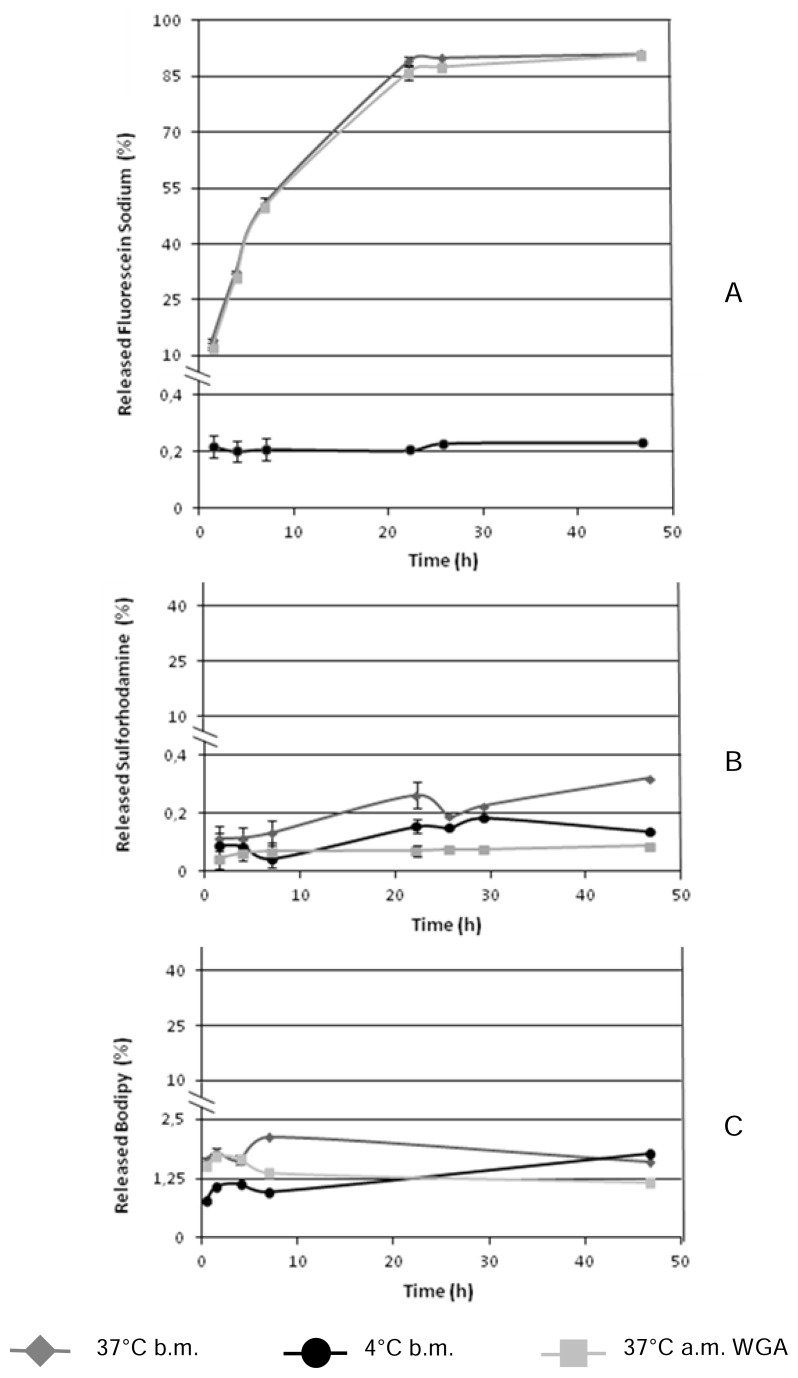
Release profiles of entrapped fluorescein sodium (A), sulforhodamine (B), and BOD (C) from PLGA microparticles. The release studies were either performed before surface modification (b.m.) at two different temperatures (4°C and 37°C), or after surface modification with WGA at 37°C (a.m. WGA) (SD ≤ 2.03, n=3).

The release studies of the entrapped fluorescent model drugs were performed before the surface modification of the microparticles at 4°C as well as 37°C and after the surface functionalization with WGA at 37°C. As demonstrated in Figure 1, in contrast to the literature [24, 25], the three types of particles exhibited no signs of first burst release which might be due to the purification steps after preparation. Interestingly, a thermo-responsive release profile of entrapped fluorescein sodium from PLGA microparticles was observed (Figure 1A) [26]. At this rate, less than 0.23% of the entrapped model drug was released from the particulate formulation after nearly 50 h incubation at 4°C in contrast to more than 90% at 37°C. However, in Figure 1B and 1C it is shown that such a thermo-responsive release from sulforhodamine- or BOD-entrapped PLGA-microparticles was not observed. According to Shive et. al. [4], PLGA microparticles smaller than 300 μm in diameter were degraded in a homogenous manner with an equivalent degradation rate at the surface and in the core. Thus, such different release profiles might be governed predominantly by two parameters: (i) the solubility of the entrapped model drugs in buffer; (ii) the diffusion velocity of the fluorescein sodium from the PLGA-matrix into the buffer. Since the inner texture of PLGA-microparticles is porous, water can easily penetrate such small holes to get contact with the encapsulated dye molecules. However, among the three types of fluorescent dyes investigated, only fluorescein sodium was excellently soluble in water and dissolved in aqueous buffer solution independently from the temperature. Therefore, the first entrapped and then dissolved fluorescein sodium easily diffused from the particle’s matrix into the surrounding medium. For the sulforhodamine-loaded microspheres, the fairly lower encapsulated amount might have led to a lower detection sensitivity of the liberated dye, and according to its amphiphilic character, its aqueous solubility was lower than that of fluorescein sodium. Finally, in the case of the BOD-loaded microspheres, the hydrophobic character of BOD favored the model drug to remain inside the hydrophobic PLGA-matrix and not to partition into the surrounding aqueous buffer. In addition, independent from the model drug, microparticles with or without WGA-grafted surfaces displayed the same release kinetics of the entrapped model drug at the same conditions.

In conclusion, due to the different properties of entrapped fluorescent model drugs as well as the influence of temperature on the PLGA-matrix, the three different types of microspheres exhibited different release profiles. In addition, particle surface modification with lectin did not affect the liberation kinetics of the entrapped model drugs.

### Cytoadhesion Studies

In order to study the cytoadhesive properties of the targeted particulate formulations, Caco-2 cell monolayers were incubated with the respective particle suspension for 30 min at 4°C. After two washing steps, the cell-associated relative fluorescence intensity was quantified. WGA is a lectin with biorecognitive properties that interacts with glycosylated structures in the intestine, which increases the bioadhesion to epithelial cells [12]. Thus, the lectin-grafted microparticles served as targeted formulations, whereas BSA- and non-grafted particles served as controls.

**Fig. 2:**
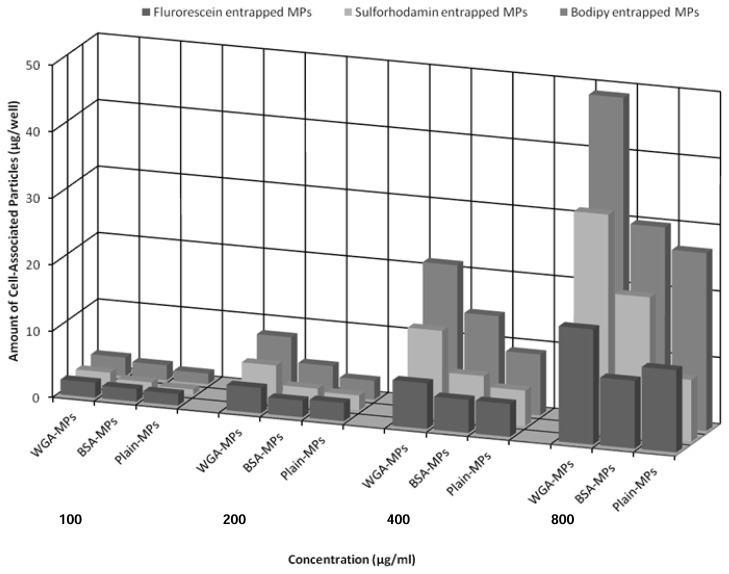
Cytoadhesion studies of fluorescein sodium, sulforhodamine, and BOD-loaded PLGA-microparticles with Caco-2 monolayers at concentrations of 100, 200, 400, and 800 μg/mL after 30 min incubation at 4°C. The surfaces of the particles were modified with WGA-, BSA-, or non-modified, abbreviated as WGA-MPs, BSA-MPs, and Plain-MPs, respectively (n = 3, SD ≤1.07).

**Fig. 3:**
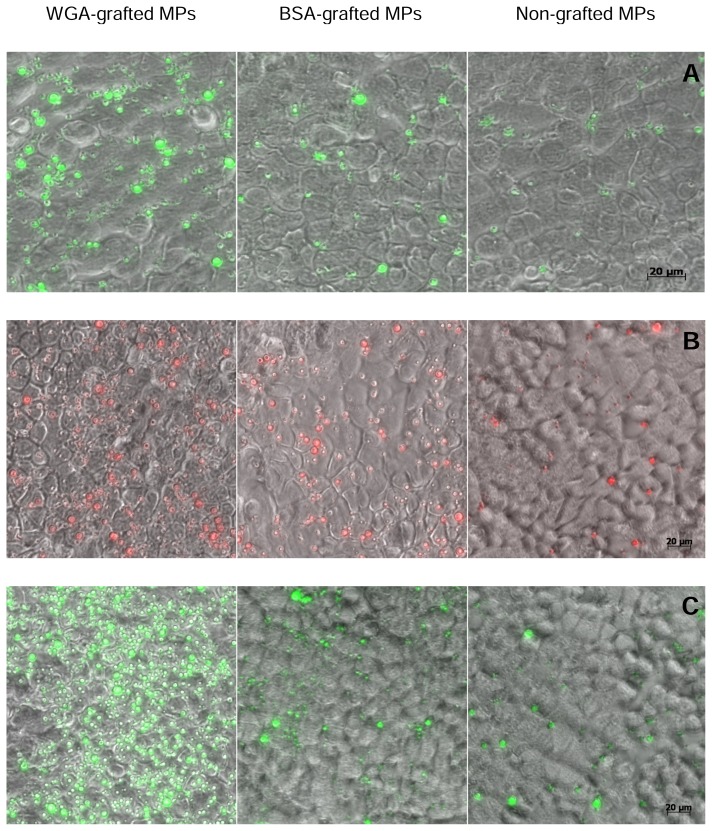
Overlay of differential interference contrast and fluorescence images of Caco-2 monolayers incubated with fluorescein sodium- (A), sulforhodamine- (B), and BOD- (C) entrapped PLGA microparticles for 30 min at 4°C followed by two washing steps (concentration of PLGA-MPs: 200 μg/mL).

As illustrated in Figure 2, independent from the type of the entrapped model drugs as well as concentrations of the applied particle suspension, the highest amount of cell-associated particles was observed in the case of the WGA-grafted microparticles exceeding that of the BSA-grafted ones up to 1.9-fold and non-grafted drug carriers 3.6-fold, respectively. These specific binding effects of WGA-functionalized particles to Caco-2 cells were also confirmed by microscopy (Figure 3). As depicted by the images, intracellular uptake of the particulate formulations was not observed. This might be due to the incubation temperature of 4°C, which inhibits energy-dependent transport mechanisms, as well as the size of the particles being too large to undergo receptor-mediated endocytosis [21]. Additionally, the amount of cell-associated particles per well increased with the concentration of applied particle suspension (Figure 2). A comparison of the surface-modified particles revealed that the BOD-entrapped microcarriers yielded the highest binding efficiency at all concentration levels, whereas fluorescein sodium-loaded particles exhibited the lowest rate of cytoadhesion. For example, the amount of cell-associated WGA-grafted, fluorescein sodium-loaded carriers ranged between 2.3±0.14 and 17.3±1.01 μg/well with a concentration of particles from 100 μg/mL to 800 μg/mL. This result corresponds to only 80–35% of that of the cell-associated BOD-entrapped microparticles at the same concentration. It should also be considered that due to the hydrophobic character of BOD as well as the amphoteric properties of sulforhodamine, which resist purification by washing with water, non-encapsulated dye might remain non-specifically adsorbed at the surface of the particles. Furthermore, such surface-anchored model drugs might detach from particles during incubation with cells to bind to the cell membrane. Importantly, such loosely bound and detached fluorescent dyes emit higher fluorescent signals in comparison to entrapped fluorescent substances inside of the particle-matrix due to quenching phenomena. Since such free fluorescent dyes can strongly contribute to the signal intensity and are also calculated as cell-bound particles, the measured amount of the cell-associated BOD- and sulforhodamine-entrapped particles might be higher than the truly cell-bound amount. Since the hydrophilicity of fluorescein anticipates the complete removal of particle-bound dye from the surface during several washing steps with aqueous buffer, such a confusing effect can be excluded. Because of this reason, the calculated amount of cell-associated fluorescein sodium particles was lower than that of the other two formulations. Interestingly, the BSA-grafted microparticles exhibited a slightly higher binding efficiency to the cell monolayer as compared to the non-grafted particles, which might be due to the nonspecific protein-protein interactions between albumin and the cell membrane.

## Conclusion

In this study, three fluorescent model drugs were microencapsulated in a hydrophobic matrix using modifications of the solvent evaporation technique according to the differing hydrophilicity of the entrapped drugs. In the case of similar hydrophobicity of both model drugs BOD and PLGA, the single emulsion technique yielded microparticles of the smallest size and highest encapsulation efficiency, but almost no release presumably due to strong hydrophobic interactions. In the case of hydrophilic fluorescein sodium and amphoteric sulforhodamine, however, only the double emulsion technique yielded microparticles. These were of larger size and lower drug loading due to the differing hydrophobicity of the drug and polymer. As amphoteric drugs and hydrophobic polymers also intensely interact, almost no release was observed from the sulforhodamine-loaded PLGA carriers. In contrast, mismatching characteristics of the drug and polymer facilitated release as indicated by the thermoresponsive release of fluorescein sodium. Consequently, the choice of the preparation technique, size, loading, as well as the release profile was basically guided by the solubility characteristics of the matrix and the entrapped drug. Moreover, non-releasing microparticles with a high quantum yield, such as the BOD-PLGA microcarriers, represent a useful tool to elucidate the interaction with cells quantitatively by fluorescence reading and qualitatively by microscopic imaging as confirmed by the Caco-2 monolayer experiments.

## Experimental

### Materials

PLGA (poly(lactic-*co*-glycolic acid), Resomer^®^ RG503H, 50:50 lactide/glycolide) was obtained from Boehringer Ingelheim (Ingelheim, Germany). PEMA (poly(ethylene-alt-maleic anhydride)), fluorescein sodium salt, sulforhodamine 101, and albumin bovine fraction V (BSA) were bought from Sigma Aldrich (Vienna, Austria). Wheat germ agglutinin (WGA) from *Triticum vulgare* was purchased from Vector laboratories (Burlingame, USA). BODIPY^®^ 493/503 (4,4-difluoro-1,3,5,7,8-pentamethyl-4-bora-3a,4a-diaza-*s*-indacene) was from Invitrogen (Eugene, USA). All other chemicals were of analytical purity.

### Preparation of Fluorescent Model Drugs Loaded with PLGA Microparticles

Sulforhodamine- and fluorescein sodium-loaded PLGA microparticles were prepared by a water-in-oil-in-water solvent-evaporation technique. For that purpose, 400 μL of an aqueous solution containing fluorescein sodium (3.75 mg/mL) or sulforhodamine (7.5 mg/mL) were emulsified with a solution containing 400 mg PLGA in 2.4 g of ethyl acetate by sonication for 2 min (sonifier: Bandelin electronic UW70/HD70; tip, MS 72/D, Berlin, Germany). After adding 8 mL of an aqueous solution of PEMA (0.5%), the emulsion was sonicated again for 2 min yielding the (w/o)/w emulsion that was poured into 100 mL of 0.25% aqueous solution of PEMA. For the BOD-entrapped microparticles, an oil-in-water solvent-evaporation technique was applied. A solution containing 400 mg PLGA and 1.5 mg BOD in 2.8 g ethyl acetate was emulsified with 8 mL of an aqueous solution of PEMA (0.5%) by sonication for 2 min yielding an o/w emulsion which was poured into 100 mL of a 0.25% aqueous solution of PEMA. After mechanical stirring at 600 rpm for 1 h at room temperature, residual ethyl acetate was removed under reduced pressure. In order to remove the non-entrapped fluorescent dyes, the microparticles were washed three times with 120 mL 20 mM HEPES/NaOH pH 7.4 each and resuspended finally in 100 mL of the same buffer. The PLGA content was determined gravimetrically from lyophilized aliquots after exhaustive dialysis against distilled water. The particle size distribution was determined by laser light scattering (Mastersizer 2000 laser particle size analyzer, Malvern Instruments, Malvern, UK).

### Surface Modification of Microparticles with Wheat Germ Agglutinin or Bovine Serum Albumin

Wheat germ agglutinin (WGA) or bovine serum albumin (BSA) was covalently bound to the surface of the PLGA microparticles following the carbodiimde method. Briefly, the surface carboxylate groups of the 5 mL PLGA microparticle suspension in 20 mM HEPES/NaOH pH 7.0 containing 0.1% Pluronic^®^ F-68 were activated by the addition of 5 mL of freshly prepared solution of 1-ethyl-3(3-dimethylaminopropyl) carbodiimide (EDAC, 1400 mg) and N-hydroxysuccinimide (NHS, 59 mg) in the same buffer. After end-over-end incubation for 30 min at room temperature, the microparticles were washed once by dilution with 15 mL of 20 mM HEPES/NaOH pH 7.4 containing 0.1% Pluronic^®^ F-68 and centrifuged (3200 rpm, 10 min, 4°C) to remove the excess reagents. For coupling, the activated microparticles were resuspended in 10 mL of the same buffer and mixed with either 500 μL aqueous solution of WGA (5 mg/mL) or 458 μL aqueous solution of BSA (10 mg/mL) by end-over-end incubation for 1 h at room temperature. Non-reacted binding sites were saturated by incubation with 2.4 mL glycine solution (100 mg/mL) in 20 mM HEPES/NaOH pH 7.4 containing 0.1% Pluronic^®^ F-68 for 30 min at room temperature. Subsequently, the microparticles were washed twice by centrifugation (3200 rpm, 10 min, 4°C) with 15 mL buffer each. Finally, the particles were resuspended in 10 mL buffer and stored at −80°C until use. As a reference, the non-grafted PLGA microparticles were treated as stated above, but by solely adding buffer instead of EDAC, NHS, WGA, BSA, and glycine.

### Determination of the Amount of Loaded Fluorescent Model Drugs

To quantify the entrapped fluorescein sodium or sulforhodamine, 300 μL aliquots of the microparticle suspensions were hydrolyzed by the addition of 100 μL 4 M NaOH and the fluorescence intensity of the 100 μL aliquots was measured at 485/525 nm (exc./em.) and 580/615 nm (exc./em.) for fluorescein sodium and sulforhodamine, respectively. The amount of the model drug was calculated from a calibration curve established with fluorescein sodium or sulforhodamine in 1 M NaOH.

To determine the amount of entrapped BOD, 150 μL aliquots of the microparticle suspensions were hydrolyzed with 50 μL of 4 M NaOH and diluted 10-fold with a mixture of DMSO and 20 mM HEPES/NaOH pH 7.4 containing 0.1% Pluronic^®^ F-68 (1+8). In 96-well microplates, 100 μL samples were analyzed at 485/525 nm (exc./em.) and the amount of the entrapped BOD was calculated from a calibration curve of BOD in a mixture of DMSO and 20 mM HEPES/NaOH pH 7.4 containing 0.1% Pluronic^®^ F-68 (1+9).

All assays were done at least in triplicate.

### Release of the Fluorescent Model Drugs from the Microparticles

To study the release kinetics of the entrapped model drugs, 500 μL suspensions in 20 mM HEPES/NaOH pH 7.4 supplemented with 0.1% Pluronic^®^ F-68, containing either plain- or surface-functionalized particles, were incubated at 4°C and 37°C. At certain time intervals, the supernatants were collected by centrifugation (14000 rpm, 5 min, 4°C) and the amount of released fluorescent dyes was determined fluorimetrically at 485/525 nm (fluorescein sodium and BOD) or at 580/615 nm (sulforhodamine). The percentage of released model drug was calculated with reference to the total dye content of the microparticles.

### Cell Cultures

The Caco-2 cell line was purchased from the German collection of microorganisms and cell cultures (DSMZ, Braunschweig, Germany). Cells were cultivated in RPMI 1640 cell culture medium containing 10% fetal calf serum, 4 mM L-glutamine, and 150 mg/mL gentamycine in a humidified 5% CO_2_/95% air atmosphere at 37°C and were subcultured by TrypLE^®^ select. Tissue culture reagents were obtained from Sigma Aldrich (St. Louis, USA) and Gibco Life Technologies Ltd. (Invitrogen Corp., Carlsbad, USA). Cells between passage 60 and 71 were used for the present study. For cell experiments and fluorescence microscopy, 1.7 x 10^4^ Caco-2 single cells were seeded per well using 96-well microplates. Cells were cultivated under standard cell culture conditions for one week to obtain a confluent monolayer.

### Cytoadhesion Studies

In order to evaluate the cytoadhesive properties of the microparticles, the particle suspensions were diluted with isotonic 20 mM HEPES/NaOH pH 7.4 to yield 100, 200, 400, and 800 μg particles/mL. After washing the confluent Caco-2 monolayers once with isotonic 20 mM HEPES/NaOH pH 7.4 (100 μL/well), the cells were incubated with 100 μL/well of the microparticle suspension for 30 min at 4°C. After removing the unbound microparticles by washing twice with isotonic 20 mM HEPES/NaOH pH 7.4 (100 μL/well), the relative fluorescence intensities were determined at 485/525 nm for fluorescein sodium and BOD or at 580/615 nm for sulforhodamine. Finally, the amount of the cell-associated particles was calculated from a calibration curve established with the particle suspensions.

### Fluorescence Microscopy

Caco-2 monolayers cultivated in 96-well microplates as described above were used for fluorescence microscopy. After washing the confluent Caco-2 monolayers once with isotonic 20 mM HEPES/NaOH pH 7.4 (100 μL/well), the cells were incubated with 100 μL of the microparticle suspension (200 μg particles/mL in isotonic 20 mM HEPES/NaOH pH 7.4) at 4°C for 30 min. Unbound particles were removed by washing the Caco-2 layers twice with isotonic 20 mM HEPES/NaOH pH 7.4 (100 μL/well). Without any further preparations, images of the cell layers were acquired using a Zeiss Axio Observer.Z1 microscopy system equipped with an LED illumination system “Colibri” (Göttingen, Germany).

## Figures and Tables

**Tab. 1:** Characteristics of the fluorescent model drug-loaded PLGA-microparticles

Type/Loading of microspheres	Preparation technique	Characteristic of dye	Mean diameter (μm)	Dye loading (μg/mg PLGA)	Encapsul. efficiency (%)
Fluorescein-Na entrapped particles/Fluorescein - Na	(w/o)w double emulsion	hydrophilic	4.5 ± 1.2	1.75 ± 0.03	46.7
Sulforhodamine entrapped particles/Sulforhodamine	(w/o)w double emulsion	amphoteric	4.7 ± 1.5	0.06 ± 0.01	0.8
BOD entrapped particles/BODIPY^®^ 493/503	o/w single emulsion	hydrophobic	4.1 ± 1.4	2.25 ± 0.07	59.9
